# Markers of Perioperative Bowel Complications in Colorectal Surgery Patients

**DOI:** 10.1155/2015/428535

**Published:** 2015-12-15

**Authors:** Radomír Hyšpler, Alena Tichá, Milan Kaška, Lenka Žaloudková, Lenka Plíšková, Eduard Havel, Zdeněk Zadák

**Affiliations:** ^1^Department of Research and Development, University Hospital Hradec Kralove, 500 05 Hradec Kralove, Czech Republic; ^2^Department of Surgery, University Hospital Hradec Kralove, 500 05 Hradec Kralove, Czech Republic; ^3^Department of Clinical Chemistry, University Hospital Hradec Kralove, 500 05 Hradec Kralove, Czech Republic

## Abstract

Colorectal cancer is a clinical condition whose treatment often involves intestinal resection. Such treatment frequently results in two major gastrointestinal complications after surgery: anastomotic leakage and prolonged ileus. Anastomotic leakage is a serious complication which, more often than not, is diagnosed late; to date, C-reactive protein is the only available diagnostic marker. A monocentric, prospective, open case-control study was performed in patients (*n* = 117) undergoing colorectal surgery. Intestinal fatty acid binding protein (i-FABP), citrulline, D-lactate, exhaled hydrogen,* Escherichia coli *genomic DNA, and ischemia modified albumin (IMA) were determined preoperatively, postoperatively, and on the following four consecutive days. Bacterial DNA was not detected in any sample, and i-FABP and D-lactate lacked any distinct potential to detect postoperative bowel complications. Exhaled breath hydrogen content showed unacceptably low sensitivity. However, citrulline turned out to be a specific marker for prolonged ileus on postoperative days 3-4. Using a cut-off value of 20 *μ*mol/L, a sensitivity and specificity of ~75% was achieved on postoperative day 4. IMA was found to be an efficient predictor of anastomosis leak by calculating the difference between preoperative and postoperative values. This test had 100% sensitivity and 80% specificity and 100% negative and 20% positive predictive value.

## 1. Introduction

Colorectal cancer is the fourth most common cause of death by cancer worldwide, whose main form of treatment consists of intestinal resection. Perioperative gastrointestinal complications or dysfunctions are frequent [1, 2]. In most cases, they are clinically suspected [3, 4] to be the result of sepsis, ileus, feeding intolerance, diarrhea, digestive bleeding, or intestinal ischemia. Evaluating the small bowel condition may in some instances be difficult for several reasons, such as its deep intra-abdominal location, nonspecific symptoms, and a lack of suitable blood plasma markers.

Two major types of gastrointestinal complications are common after colorectal surgery: anastomotic leakage (dehiscence) and prolonged ileus. Of particular concern are the complications by anastomotic dehiscence occurring mainly after rectal surgery. It is often diagnosed late due to a low index of suspicion based on clinical and conventional laboratory practice [5]; often leading to the development of septic complications and reoperation. Elevated C-reactive protein levels have been found as an “early” indicator of anastomotic leakage from the second to fourth postoperative days. However, it offers rather poor sensitivity and specificity (70–80%) [6] and usually reflects an already-triggered inflammatory reaction.

As correctly stated by Steele et al. [3], the major contributing factor to the viability and integrity of an anastomosis procedure is adequate blood flow, suggesting that anastomosis ischemia is probably the leading cause of dehiscence. Recently, a new test for ischemia-modified albumin (IMA) detection was developed using copper binding assay [7]. This modified assay offers several advantages over the older and widely used cobalt binding assay [8]. The copper binding assay possesses improved sensitivity on the albumin's N-terminal aminoacid residues' capacity for binding transition metal ions. IMA has been tested as a potential marker of bowel ischemia, as detected by cobalt binding assay, with some level of efficiency [9, 10]. It is sensitive enough to detect small areas of bowel ischemia during hernia incarceration in animal experiments [11].

Intestinal ischemia also causes epithelial destruction, which has been associated with an elevated plasmatic concentration of i-FABP (Intestinal Fatty Acid Binding Protein), a protein expressed in the cytosol of enterocytes and one of the proposed markers of enterocyte necrosis [12]. The major disadvantage of i-FABP, as a highly organ specific protein, is the desquamation of enterocytes into the lumen when they are damaged and the consequent limited absorption of the protein into the body. This is probably the reason for the poor sensitivity of the analysis described in available meta-analyses [12].

Plasmatic D-lactate concentration has been proposed as a sensitive marker in detecting gut failure and endotoxemia likely due to an impaired intestinal barrier function [13]. It is normally produced in the fermentative organs of the gastrointestinal tract (cecum, colon). Several pathogenic bacteria produce D-lactate, including* Bacteroides fragilis*,* Escherichia coli*,* Klebsiella pneumoniae*, and* Staphylococcus aureus*. Gut ischemia results in elevated D-lactate levels [13], a condition also found in peristaltic disorders such as blind loop syndrome. A similar test, the quantization of hydrogen found in exhaled breath as a marker of* Klebsiella* and* Staphylococcus* overgrowth in the bowel lumen has also been attempted [14].

Gastrointestinal dysfunction due to a prolonged ileus has been associated with a worse prognosis in postoperative patients, particularly when persisting for more than one week [15]. According to the European Society for Clinical Nutrition and Metabolism recommendations [16], prolonged postoperative ileus is a type I intestinal failure and of spontaneously resolving nature. Currently, enhanced recovery techniques are available, although a suitable laboratory marker is still lacking.

Citrulline is an *α*-amino acid synthesized mainly from glutamine by small bowel enterocytes [17]. Plasmatic citrulline concentration (normally within 20–40 *μ*mol/L) is determined by the balance between gut citrulline synthesis and kidney citrulline degradation. It has been previously demonstrated that plasmatic citrulline concentration is a simple and reliable biomarker of enterocyte mass in patients with chronic small bowel pathologies. It can be correlated with the severity and extent of villous atrophy in both patients with normal small bowel length or villous atrophy associated small bowel disease [18].

On a different subject, bacterial translocation is defined as the escape of viable indigenous bacteria from the gastrointestinal (GI) tract into the mesenteric lymph nodes, liver, spleen, and bloodstream. In this regard, PCR analysis has a higher sensitivity than blood or mesenteric lymph node cultures in assessing bacterial translocation (most commonly* E. coli*) from the intestine during an early postabdominal surgery stage [19].

The present study aims to develop and validate suitable, readily available, and relatively noninvasive biomarkers for the diagnosis and monitoring of postoperative gastrointestinal complications, where early diagnosis is of utmost importance. Due to the unavailability, or nonexistence, of other diagnostic methods, laboratory tests from blood samples are the definitive candidates as diagnostic tools of small bowel damage evaluation. Ischemia-modified albumin was preliminarily found by us as a very promising predictor of anastomosis leakage, already showing positive results in as little as two hours after the operation. On the other hand, citrulline could be used as a suitable monitoring marker of prolonged postoperative ileus. As this manuscript is being written, any of these findings have not yet been published.

## 2. Patients and Methods

A monocentric, prospective, open case-control study was performed in patients undergoing colorectal surgery. The study focused primarily on the identification of plasmatic biomarkers of bowel postoperative complications. All procedures were approved by the Ethics Committee (Ref. number 201107S45P) according to the Declaration of Helsinki (June 1964 and later amended). Eligible patients were properly informed of the study's aim and methods in both verbal and written form, and a willing informed consent was obtained.

The inclusion criteria encompassed elective large bowel surgery for malignant disease, involving one resection of the pathological bowel segment and one anastomosis, and the absence of any other primary bowel disease (such as Crohn disease or ulcerative colitis), severe nephropathy, or hepatopathy. The mean time duration of operation was of 160 ± 65 min and operational trauma did not vary significantly within individual cases.

The study protocol consisted of blood sample collection (8 mL of peripheral blood into a BD Vacutainer with dipotassium EDTA) preoperatively (day −1), early postoperatively (120 ± 30 min after the end of the operation, day 0), and daily postoperatively for four consecutive days (6-7 a.m., days 1–4). Blood samples were afterwards analyzed by the methods further described.

*Intestinal fatty acid binding protein (i-FABP)* was analyzed using the Human i-FABP ELISA kit (Hycult Biotech, Netherlands) according to the manufacturer's instructions.

*Citrulline plasmatic concentration* was analyzed using high performance liquid chromatography with the addition of fluorescence detection (Shimadzu LC-10A, Shimadzu, Japan), after plasma ultrafiltration (Amicon 10 kDa, Merck, Germany) and derivatization using* o*-phthalyldialdehyde and mercaptopropionic acid [20]. The fluorescence detector was set at excitation/emission wavelengths of 235/340 nm. Chromatographic separation was carried out in a Merck LichroCART 250x4 Lichrospher RP-18e, 5-micron column.

*D-Lactate plasmatic concentration* was determined by an in-house developed protocol [21] using deproteination by ultrafiltration (Amicon 30 kDa, Merck, Germany), followed by the coupled reaction of D-lactate dehydrogenase, to reduce nicotinamide adenine dinucleotide, and D-glutamate pyruvate transaminase. The reagents were obtained from a D-lactate assay kit manufactured by Megazyme International (Wicklow, Ireland). The end-point concentration of nicotinamide adenine dinucleotide was determined by measuring absorbance at 340 nm.

*Hydrogen concentration from exhaled breath* was evaluated using a Gastro+ analyzer (Bedfont Scientific Ltd., UK). The instrument was calibrated and validated according to manufacturer's instructions using the original gas mixture.

*E. coli genomic DNA content* was analyzed in 1 mL of plasma by QIAamp DNA Mini Kit (Qiagen, Hilden, Germany). Primers were designed targeting the uidA gene (beta-glucuronidase gene specific for* E. coli*) and amplified by nested PCR [22]. Negative results were confirmed by two methods of quantitative DNA determination. Firstly, real-time PCR was carried out using primers targeting the sfmD gene area coding “a putative outer membrane export usher protein” [23]. Secondly, in-house primers and probe (TaqMan format) from* E. coli* 16S ribosomal RNA gene (GenBank Accession number J01859.1) were designed as follows: sense primer 5′-GGTAGAATTCCAGGTGTA-3′, antisense primer 5′-GGGTATCTAATCCTGTTTG-3′, and probe FAM-TGAGCGTCAGTCTTCGTCCA-BHQ1. TaqDNA inhibition was checked in all samples with negative results. Increased PCR sensitivity by preamplification was checked, with no apparent influence on the results obtained.

*Plasmatic concentation of Ischemia modified albumin* was determined by copper binding assay as previously described [7], using a microplate reader Infinite 200 PRO (Tecan, Switzerland). The protein fraction was purified from interfering low-molecular weight substances by ultrafiltration (Amicon 30 kDa, Merck, Germany) followed by double wash with phosphate buffered saline and then mixed with copper (II) chloride for 10 minutes. The residual, unbound copper was determined by fluorescence quenching of lucifer yellow dye.

## 3. Results

Operation trauma had a significant effect on all the parameters tested. Out of 117 enrolled patients (Table 1), complication cases were recorded in 25 (21.4%) patients (Table 2). Twelve complication cases (10.3%) were directly related to the patients' bowel, with prolonged ileus and anastomosis leakage complications being predominant. The complication rate was found to be dependent upon tumor location. Of the number of patients with colon tumor (*n* = 50) only 2% (1 case) suffered from prolonged ileus, and another 2% (1 case) suffered from anastomosis leak; however, the patients with rectal tumor (*n* = 67), showed a higher rate of postoperative complications: 6% (4 cases) suffered from prolonged ileus and 6% (4 cases) from anastomosis leak.

The results obtained from the analysis of potential markers are shown, with their statistical significance, and compared to baseline values in Table 3.

Genomic DNA from* Escherichia coli* was not detected in any sample despite great effort dedicated to improve the sensitivity of the analysis.

The plasma concentration level of i-FABP was found to be increased at early stages of the analysis (day 0), only to decrease significantly at a later postoperative period (day 3-day 4). This marker was unable to discriminate between the studied bowel complications despite it being an organ specific protein. D-Lactate plasmatic concentration, however, was found in significantly elevated levels throughout the studied postoperative period and did not return to baseline values even after four days. This marker, though, also lacked the capacity to further distinguish between the studied bowel complications. Hydrogen content from exhaled breath, another analyte related to bacterial metabolism, was also found in increased levels within the postoperative period. Its higher concentrations were correlated with an elevated incidence of postoperative complications. A preselected cut-off value of 10 ppm showed sensitivity/specificity rates of 41.7%/88.6% for bowel complications and 35.7%/94.9% for extrabowel complications. An increased cut-off value of 20 ppm resulted in sensitivity rates of 33.3% and 28.6% and specificity of 98.1% and 98%, respectively.

**Figure 1:**
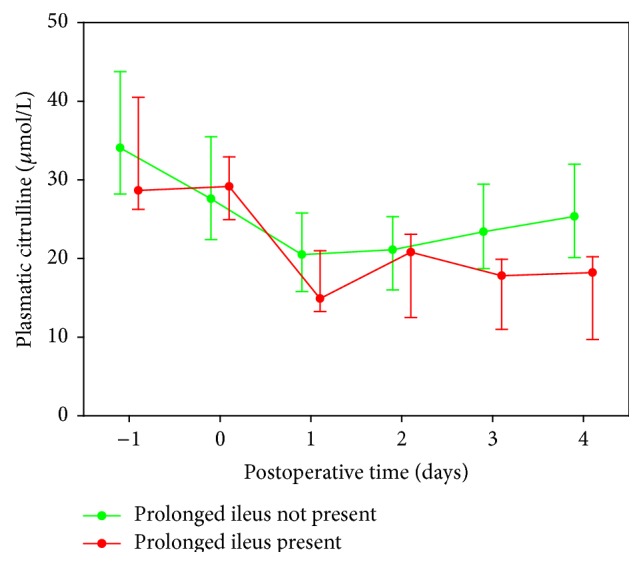
Time-profile of citrulline concentration in plasma.

**Figure 2:**
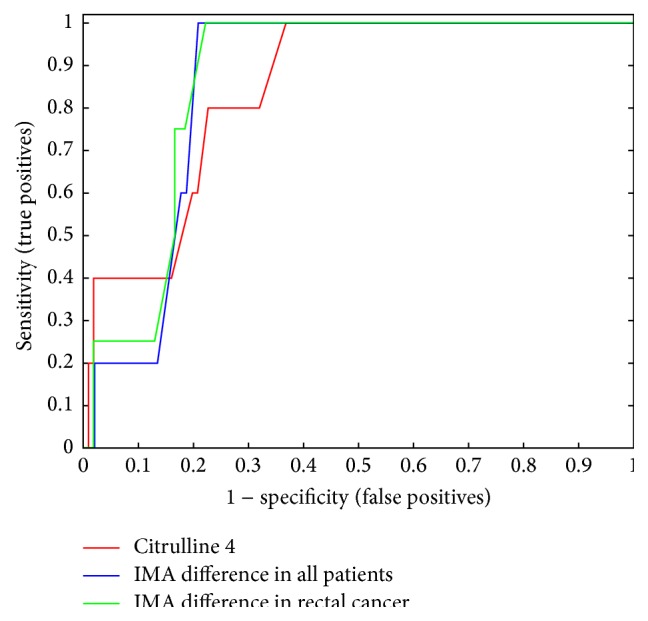
ROC curves of citrulline versus prolonged ileus (in all patients) and IMA difference versus anastomosis leak (in all patients and in colorectal cancer patients).

**Figure 3:**
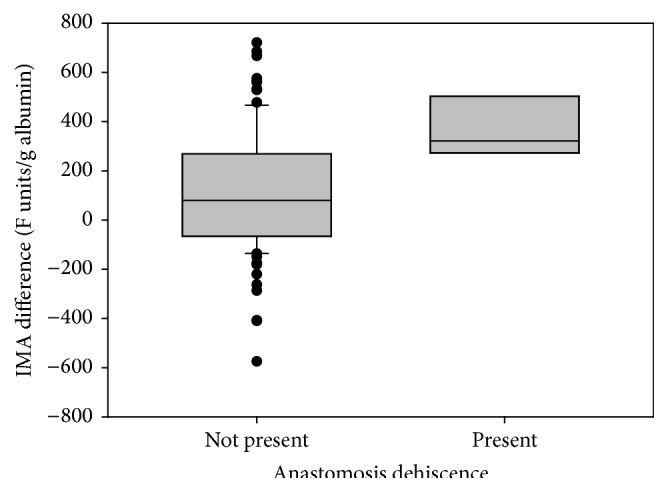
IMA difference (preoperative-postoperative value) versus anastomosis leak (*p* = 0.017).

Citrulline plasmatic levels were significantly decreased during the postoperative period, reflecting a diminished enterocyte metabolic activity. Citrulline, however, turned out to be a specific marker for prolonged ileus on postoperative days 3-4 (Figure 1). A cut-off value of 20 *μ*mol/L achieved sensitivity and specificity rates of 75% and 76%, respectively, in the diagnosis of prolonged ileus on postoperative day 4 (Figure 2). At first glance, plasmatic IMA concentration levels did not seem to deviate significantly from preoperative to postoperative periods within the analyzed patients' group and, due to its relatively short half-life, we considered it unnecessary to further analyze its plasmatic level at later stages of the postoperative period. Regardless, plasmatic IMA concentration difference values (postoperative minus preoperative) were calculated seeking to eliminate the known large biological scatter and, surprisingly, we found IMA to be an efficient predictor of anastomosis leak (Figure 3, *p* = 0.017) (ROC curves for IMA as a predictor of anastomosis leak are presented in Figure 2). Assuming a difference of 270 fluorescent units per gram of albumin as a cut-off value, the test had 100% sensitivity and 80% specificity, meaning a 100% rate of negative predictive value and 20% positive predictive value.

## 4. Discussion

The results obtained in the present study demonstrate some of the existent pitfalls in the interpretation of bowel diseased states as given by the sole analysis of a biomarkers' change patterns. The bowel specific markers i-FABP and D-lactate, which are significantly increased in mesenteric ischemia, proved to be clinically useless in the prediction or monitoring of postoperative complications. Concerning i-FABP, the lack of diagnostic efficiency could be attributed to an insufficient amount of enterocytes undergoing perioperative necrosis, although the postoperative time-profile could also be considered partly at fault since the significant decreased levels of plasmatic i-FABP were probably caused by a diminished enterocyte turnover during the selected time period for the analysis.

D-Lactate, predominantly a byproduct of gut flora metabolism, was found in increased levels during the postoperative period, most likely due to unbalanced bowel microbiota populations caused by prophylactic antibiotics and preoperative bowel preparation. Exhaled breath hydrogen content, another microbial byproduct, exhibited a better outcome in the detection of perioperative complications, further assessing a defined role for bowel microbiota metabolic byproducts' as a diagnostic tool of postoperative complications. Its apparent usefulness notwithstanding the proposed method suffers from too low sensitivity to be of any clinical value.

The most disappointing results were obtained from the* E. coli* genomic DNA detection test. Despite optimistic references [19], the protocol, as employed by our group, did not manage to detect any* E. coli* genomic DNA in the patients' plasma. The sensitivity of the test has been determined in ~10 DNA copies per milliliter of plasma. It is rather possible that the operational procedure for colorectal cancer, according to local standards, does not cause significant bacteremia.

Of all the included and tested markers, only two were found of any clinical value: citrulline and IMA. Citrulline was defined as a suitable marker of postoperative prolonged ileus because its plasmatic concentration reflects the metabolic and peristaltic activity of the bowel. Decreased basal values of plasmatic citrulline concentration were found in all the analyzed patients, reflecting a “physiological” postoperative ileus status. Afterwards, these concentration values were increased in the absence of prolonged postoperative ileus and remained low in its continued presence. Therefore, we suggest that plasmatic citrulline concentration could be used clinically even without the knowledge of basal values. Its reference interval ranges from 20 to 40 *μ*mol/L, with 20 *μ*mol/L as a suitable cut-off value for prolonged ileus testing. Citrulline is usually analyzed as part of the amino acid spectrum by high performance liquid chromatography thus representing a major drawback in its clinical use. Citrulline content analysis is only available in large hospitals, and same-day laboratory response is hard to achieve, if not unfeasible. Also, as prolonged ileus is commonly a self-resolving condition [16], there is little clinical demand for a molecular diagnostic biomarker. Regardless, citrulline still may be useful as a potential marker in research studies dealing with preoperative or postoperative changes, enabling the quantitative estimation of prolonged postoperative ileus severity.

The major finding of this study was the clinical value of IMA in diagnosing postoperative complications of colorectal surgery. Anastomosis leakage is a potentially disastrous condition which often leads to sepsis and postponed adjuvant chemotherapeutic treatment, therefore compromising the survival rate of the patients. C-reactive protein has been previously identified as the optimal marker available, yet it can only be used until the fourth postoperative day with a set cut-off value of 150 mg/L [24]. However, it is a marker indicating an already ongoing inflammatory response; thus an earlier marker is needfully sought after [2, 3, 24]. The major role of ischemia in anastomosis leak has been acknowledged [2], but a suitable early marker has not yet been identified. Cobalt-based IMA assays were used as early as the year 2000 [8] and, for approximately ten years after that, as a marker of coronary events but it could not claim superiority over other previously well-established cardiomarkers and its clinical use has recently vanished. Its characteristics as a sensitive and nonspecific organ marker, along with an improved and quantifiable detection method by copper-binding assays, make it useful in the early diagnosis of perioperative bowel damage. As it is known from cobalt-based assays, IMA suffers from large interindividual variability, which may be overcome by pre- and postoperative determination. Also, there is room for potential overall improvement of the assay as well. Copper binding to albumin molecules is known to be pH dependent [25]. The developed assay (CuBA, [7]) omitted any buffering, despite the tendency of serum pH towards the alkaline range (approx. pH 8) due to CO_2_ loss* ex vivo*. In alkaline solution, albumin binds Cu(2+) ions not only to N-terminal site, but also to the metal binding site [25], which would lead to a higher biological variability in the test. This concerning issue was solved by implementing a double protein wash of plasma proteins with phosphate buffered saline using ultrafiltration.

The theoretical outcome of the CuBA test, as currently developed, is outstanding [7] though still in need of validation in human clinical samples and it could also benefit from further analytical improvements. Some additional advantages of the test lie in its simplicity and low economical cost. Nevertheless, these significant results should be independently assayed and confirmed on a higher number of patients, further verifying sensitivity, specificity, negative and positive predictive values, and accuracy of the test.

## 5. Conclusions

The present study achieved the novel identification of two clinically useful marker tests for oncological colorectal surgery complications. The *α*-amino acid citrulline may well be of clinical assistance in distinguishing between prolonged ileus and postoperative metabolic “stunning” of the small bowel. On the other hand, ischaemia modified albumin has been proved as an effective predictor of anastomosis leakage, which could be useful in the early detection of this most serious periperative complication common amongst rectal cancer patients.

## Figures and Tables

**Table 1:** Study group description (number of cases are shown if not otherwise stated).

Characteristic	Number of cases
Total number of patients	117
Sex	67 m, 50 f
Age (years)	66 ± 9.5
Body mass index (kg·m^−2^)	26.9 ± 4.8
ASA physical status classification	81 (II), 35 (III), 1 (IV)
Localisation of tumor	50 colon, 67 rectum
Operation duration (min)	160 ± 65
Hypertension comorbidity	71
Diabetes comorbidity	34
Coronary artery disease comorbidity	15
Obesity	29
Neoadjuvant chemoradiotherapy	39
Intensive care unit stay (days)	1.6 ± 1.4
Hospital stay (days)	10.5 ± 6.7
First postop gas passage (days)	1.8 ± 1.0
First postop stool passage (days)	2.8 ± 1.6

**Table 2:** Complications.

Complication group	Complication type	Number of cases
Extrabowel related	Cardiac insufficiency	3
	Renal insufficiency	1
	Wound dehiscence, infection	5
	Refeeding syndrome	1
	Bronchopneumonia	2
	Pancreatitis	1

Bowel related	Anastomosis leak	5
	Prolonged ileus	5
	Bleeding intraluminal	1
	Abscess	1

**Table 3:** Laboratory markers.

Test	Day −1	Day 0	Day 1	Day 2	Day 3	Day 4
Hydrogen (ppm)	ND	1 (0, 1) max. 14	1 (0.75, 2) max. 12	1 (1, 3) max. 33^*∗*^	1 (1, 4) max. 28^*∗*^	1 (1, 3) max. 29^*∗*^

i-FABP (pg/mL)	417 (232, 748)	583^*∗∗*^ (318, 965)	462 (292, 762)	367^*∗*^ (162, 563)	269^*∗∗*^ (163, 432)	284^*∗∗*^ (143, 519)

D-Lactate (*μ*mol/L)	33.4 (26.3, 39.7)	90.2^*∗∗*^ (78.0, 102)	112^*∗∗*^ (79.1, 139)	95.6^*∗∗*^ (46.9, 137)	48.3^*∗∗*^ (32.6, 73.8)	35.8^*∗∗*^ (28.4, 48.7)

Citrulline (*μ*mol/L)	34.0 (28.1, 43.7)	27.6^*∗∗*^ (22.6, 35.4)	20.2^*∗∗*^ (15.3, 25.1)	21.1^*∗∗*^ (16.0, 24.9)	22.8^*∗∗*^ (18.5, 29.3)	24.9^*∗∗*^ (19.1, 32.0)

IMA (F units/g albumin)	799 (653, 892)	740 (498, 881)	ND	ND	ND	ND

^*∗*^ *p* < 0.05 against baseline value, ^*∗∗*^ *p* < 0.001 against baseline value.

Data are presented as median (25th, 75th percentile).

ND: not determined.
